# Patient-reported outcomes during accelerating the en-masse retraction of the upper anterior teeth using low-intensity electrical stimulation: a randomized controlled trial

**DOI:** 10.1186/s40510-024-00517-3

**Published:** 2024-05-13

**Authors:** Rashad I. Shaadouh, Mohammad Y. Hajeer, Ghiath A. Mahmoud, Imad Addin Almasri, Samer T. Jaber, Mohammad Khursheed Alam

**Affiliations:** 1 Department of Orthodontics, Faculty of Dentistry, University of Damascus, Damascus, Syria; 2 Department of Applied Statistics, Faculty of Economics, University of Damascus, Damascus, Syria; 3 Department of Orthodontics, Faculty of Dentistry, Al-Wataniya Private University, Hama, Syria; 4 Orthodontic Division, Preventive Dentistry Department, College of Dentistry, Jouf University, Sakaka, 72345 Saudi Arabia

**Keywords:** En-masse retraction, Low-intensity electrical stimulation, Pain, Satisfaction, Visual analog scale

## Abstract

**Background:**

Low-intensity electrical stimulation (LIES) is considered a relatively recent technology that has received little attention in orthodontics as a method of acceleration. This study aimed to evaluate patient-reported outcome measures when LIES is used to accelerate the en-masse retraction of the upper anterior teeth.

**Materials and methods:**

The sample consisted of 40 patients (8 males, 32 females; mean age 21.1 ± 2.3 years), with Class II division I malocclusion who required extraction of the first premolars to retract upper anterior teeth. They were randomly assigned to the LIES group (*n* = 20) and the conventional en-masse retraction group (CER; *n* = 20). Patient responses regarding pain, discomfort, burning sensation, swelling, chewing difficulty, speech difficulty, and painkillers’ consumption were recorded at these nine assessment times: 24 h (T1), 3 days (T2), and 7 days (T3) after force application, then in the second month after 24 h (T4), 3 days (T5), and 7 days (T6) of force re-activation, and finally after 24 h (T7), 3 days (T8), and 7 days (T9) of force re-activation in the third month.

**Results:**

The mean values of pain perception were smaller in the LIES group than those in the CER group at all assessment times with no statistically significant differences between the two groups except during the second and third months (T5, T6, T8, and T9; *P* < 0.005). However, discomfort mean values were greater in the LIES group with significant differences compared to CER group during the first week of the follow-up only (T1, T2, and T3; *P* < 0.005). Burning sensation levels were very mild in the LIES group, with significant differences between the two groups at T1 and T2 only (*P* < 0.001). Speech difficulty was significantly greater in the LIES group compared to CER group at all studied times (*P* < 0.001). High levels of satisfaction and acceptance were reported in both groups, without any significant difference.

**Conclusion:**

Both the LIES-based acceleration of en-masse retraction of upper anterior teeth and the conventional retraction were accompanied by mild to moderate pain, discomfort, and chewing difficulty on the first day of retraction. These sensations gradually decreased and almost disappeared over a week after force application or re-activation.

**Trial registration:**

ClinicalTrials.gov, ClinicalTrials.gov, NCT05920525. Registered 17 June 2023 - retrospectively registered, http://clinicaltrials.gov/study/NCT05920525?term=NCT05920525&rank=1.

## Introduction

Orthodontic pain is an unpleasant feeling that is associated with various orthodontic procedures such as elastic separator placement [1], orthodontic wire insertion and activation [2], impacted canines traction [3], anterior teeth retraction [4], and functional and removable appliance using [5, 6]. The forces applied by orthodontic appliances during teeth moving trigger a specific inflammatory response in the periodontal ligament and result in the successive release of biochemical mediators like prostaglandins, bradykinin, and histamine and vascular occlusion, which result in pain perception by stimulating local nerve endings [7–9]. According to many surveys, pain was considered one of the major barriers to receiving orthodontic treatment, had a bad effect on patient’s compliance, and was a cause of treatment discontinuation or appointment non-commitment [10]. Therefore, pain management becomes a critical factor for successful treatment.

Furthermore, the long duration of the orthodontic correction is another main challenge that may discourage patients from undergoing this treatment [11]. The average duration of orthodontic treatment ranges between 18 and 24 months [12]. This long-lasting period may reduce patient acceptance and cooperation and increase the risk of developing white spots, caries, gingival recession, and root resorption [13, 14]. Therefore, researchers and clinicians have sought to reduce the orthodontic treatment time and enhance the rate and efficacy of orthodontic tooth movement [15].

Numerous surgical and non-surgical techniques have been tested as a means of speeding up orthodontic treatment [16]. Although surgical techniques such as corticotomy [17], laser-assisted flapless corticotomy [15], periodontally accelerated osteogenic orthodontics (PAOO) [18], distraction osteogenesis [19], corticision [20], piezocision [21, 22], and micro-osteoperforations [23] have been shown to be effective in accelerating teeth movement with many different patterns of malocclusion. However, these techniques may be undesirable by patients due to anxiety and fear of pain and surgery [24]. On the other hand, low-level laser therapy (LLLT) [25], resonance vibration [26], low-intensity pulsed ultrasound [27], and pulsed electromagnetic fields [28] were tested as non-surgical techniques in order to avoid the negative effects of the surgical techniques. Furthermore, some studies suggested that many of these physical techniques could alleviate pain related to orthodontic treatment [29, 30].

Another promising modality to accelerate orthodontic tooth movement (OTM) is microcurrent electrical therapy (MET), which is considered a relatively recent technology that has received little attention in dentistry [31]. Several animal studies that evaluated the effect of electric current on orthodontic treatment indicated that with a combination of electrical current and mechanical force, teeth moved much faster than those treated with orthodontic forces alone [32, 33].

However, the main problem that prevented the use of electrical stimulation to accelerate orthodontic tooth movement was how to apply it inside the mouth [34]. A clinical study by Kim et al. on seven female patients found that the use of an external micro electric current from a small electrical device may speed up the orthodontic movement by one-third, this may reduce the duration of orthodontic treatment [35]. Also, another pilot clinical trial was conducted by Shaadouh et al. who used a new electrical removable device that applied low-intensity electrical stimulation during the en-masse retraction of the upper anterior teeth. They reported that the application of LIES on the anterior region of the maxilla for five hours daily was an effective method to increase the retraction rate of the upper anterior teeth with an average rate of 0.97 ± 0.06 mm/month [36].

However, to judge the suitability of this technique in clinical practice, it is not enough to study the effectiveness and feasibility of this method only. The patient-centered outcomes such as pain, discomfort, and satisfaction accompanying the use of this method should also be evaluated. Furthermore, the extent of patients’ cooperation and acceptance of this method must be assessed [11, 37]. Therefore, the current study aimed to evaluate pain, discomfort, swelling, difficulties of mastication, speech difficulties, and patient satisfaction levels accompanying the application of a micro direct electric current during the en-masse retraction of the upper anterior teeth using a portable device that was specifically created to provide the necessary electric stimulation.

## Materials and methods

### Trial design, registration, and post-trial registration changes

This trial was a randomized clinical trial with a single-blind, two-arm parallel groups design with an allocation ratio of 1:1. This trial was registered at the Clinical Trials database (https://clinicaltrials.gov; NCT05920525). The study was approved by the Local Research Ethics Committee of the University of Damascus (UDDS-594-09032021/SRC-2735). No change occurred in the research protocol after the trial registration.

### Sample size estimation

The Minitab® Version 18 program (Minitab Inc., State College, Pennsylvania, USA) was used to calculate the sample size based on a similar study [38]. With a significance level of 5% and a study power of 85%, assuming that the least significant difference desired to be detected between both groups in the pain level was 1.5 cm on the visual analog scale (VAS). The standard deviation was 1.5 from the previous study [38]. The required sample size was found to be 38 patients, 19 patients in each group. An additional patient was added to each group to compensate for any possible withdrawal.

### Study settings, participants, and inclusion criteria

Participants were chosen from the patients attending the Department of Orthodontics in the Faculty of Dentistry at Damascus University during the period between March 2021 and September 2022. A total of 320 patients were examined by the researcher, and 50 patients were selected based on the intra- and extra-oral clinical examination and radiographic evaluation, and who met the following eligibility criteria: adults between the ages of 17 and 26 years, Class II Division 1 malocclusion according to Angle’s classification, which required the extraction of the upper first premolar only as a part of the orthodontic treatment plan to cover up the case, mild to moderate skeletal class II (ANB = 5–7), protrusion less than 10 mm (5 to 10 mm of overjet), normal or vertical growth pattern (MM ≥ 26; SN-MP ≥ 33; Y Axis ≥ 65), normal or shallow overbite, dental crowding less than 3 mm, the presence of all permanent upper teeth (regardless of third molars), good oral health (probing depth ≤ 3 mm; plaque and gingival index ≤ 1), no general health condition that affects dental movement rate, and no previous orthodontic treatment.

**Fig. 1:**
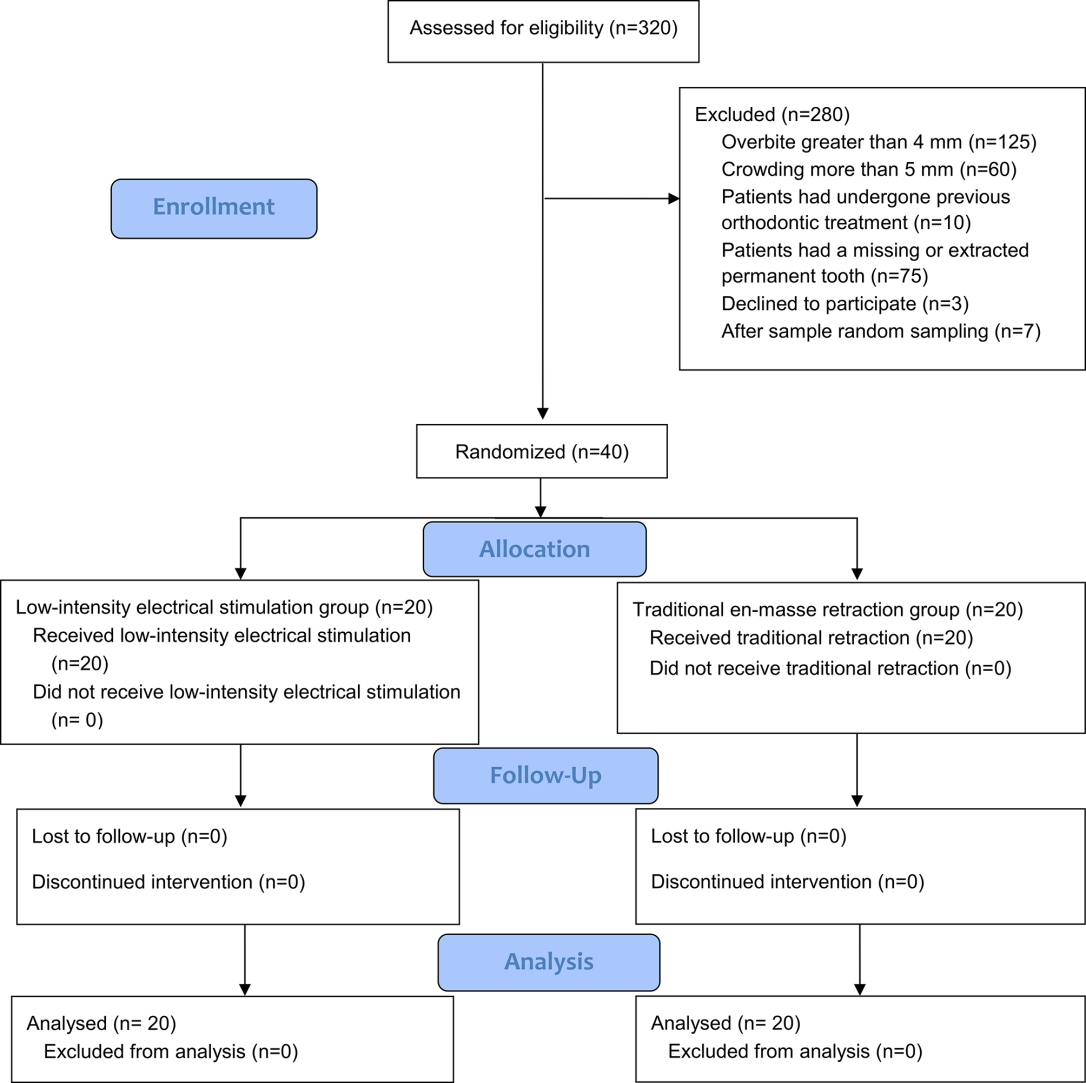
Consort flow diagram

After providing them with an information sheet, informed consent was obtained from the 47 patients who agreed to participate in the study. These patients were then assigned serial numbers from 1 to 47. Forty patients were randomly selected by Minitab® Version 18 and included in the study. The Consolidated Standards of Reporting Trials (CONSORT) flow diagram of patient recruitment, follow-up, and entry into data analysis was given in Fig. 1.

### Randomization, blinding, and allocation concealment

Patients were randomly divided into 2 groups: the low-intensity electrical stimulation group (LIES group; *n* = 20), and the conventional en-masse retraction group (CER group; *n* = 20), which served as a control group. Simple randomization was carried out using computer-generated random numbers with an allocation ratio of 1:1. The allocation sequence was concealed by using sequentially numbered, opaque, sealed envelopes that were opened only after the leveling and alignment stage was completed. Patient and researcher blinding was not applicable. Therefore, blinding was applied only during outcome assessment.

### Conventional en-masse retraction group

Leveling and alignment process was performed using pre-adjusted fixed orthodontic appliances of 0.022 × 0.028-inch slot metal bracket with MBT™ prescription (Votion™, Ortho Technology, Florida, USA), The conventional sequence of wire replacement was followed until a 0.019 × 0.025 Stainless Steel base wire was attached. After three weeks, this wire was replaced with a wire of the same type and size, equipped with two hooks of 8–10 mm height distal to the canines.

The maxillary first premolars were extracted, and skeletal anchoring was applied before leveling and alignment began for all patients. Self-drilling orthodontic mini-implants (diameter, 1.6 mm; length, 8 mm; 3 S screw, Hubit, Seoul, Korea) were placed between the roots of the maxillary second premolar and the first molar on each side.

**Fig. 2:**
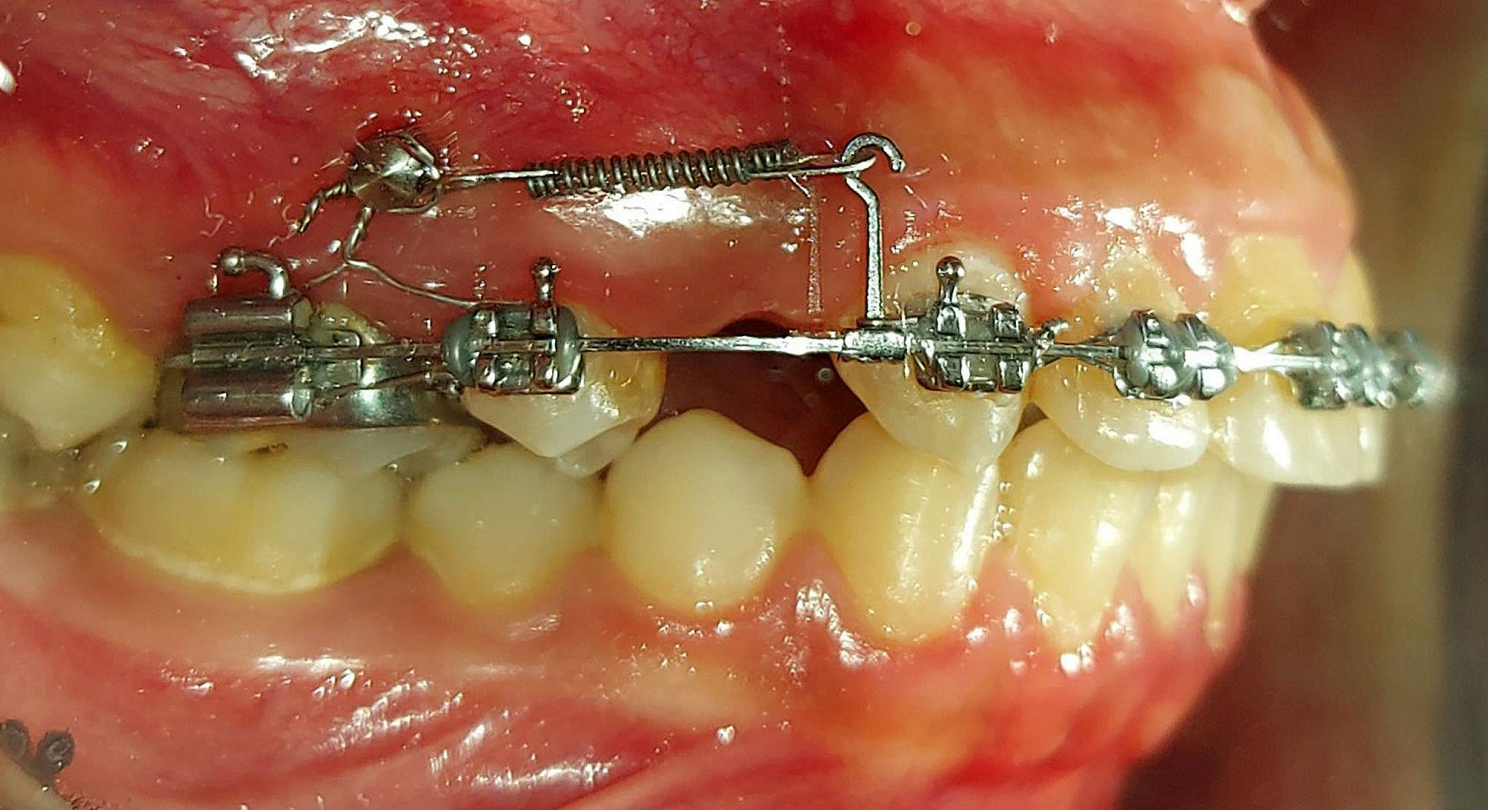
The conventional miniscrew-based en-masse retraction of upper anterior teeth

The en-masse retraction was performed using nickel-titanium (NiTi) closed coil springs (NT3 closed coil, American Orthodontics, Sheboygan, Wis). These springs were stretched from the hooks to the mini-implants and applied 250 g of force per side (Fig. 2). The force was examined using a force gauge at every appointment (040-711-00; Dentaurum, Ispringen, Germany).

Patients’ follow-up visits were scheduled every 15 days. Each appointment involved calibrating and, if necessary, readjusting the force. The retraction phase was considered over when the canines get into a class I relationship with regular overjet.

### Low-intensity electrical stimulation group

**Fig. 3:**
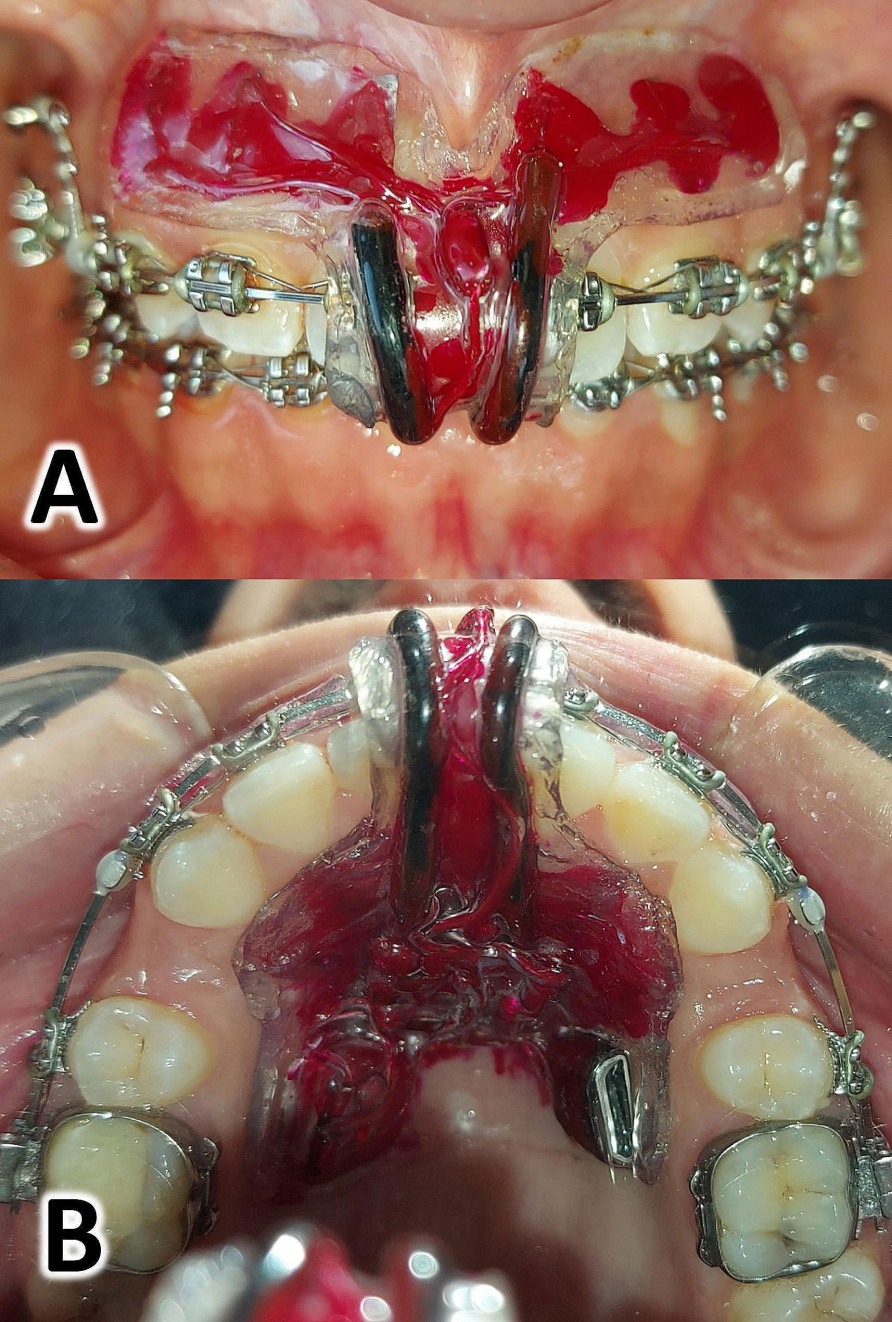
The electrical stimulation device **A**: Frontal view of the device inside the patient’s mouth; **B**: Occlusal view of the device inside the patient’s mouth

After completing the leveling and alignment stage as described in the control group (the CER group), and before starting the en-mass retraction stage, alginate impressions were taken for all patients in the experimental group. These impressions were used to fabricate the electrical accelerator device for each single patient (Fig. 3).

After testing the device inside the mouth and and ensuring that the resulting current parameters (intensity and voltage) were correct, and the absence of any other problems such as irritation of the periodontal tissues or the lips, each patient was instructed to wear the device inside the mouth for 5 h a day. Patients were asked to use the electrical device in conjunction with the application of the en-mass retraction forces until completion of the upper anterior teeth retraction.

### The electrical accelerator device

The electrical accelerator device used in this trial was designed by the researchers RIS and MYH to provide the electrical stimulation. It was a removable device fitted in the anterior region of the patient’s maxilla and was designed to apply a direct, low-intensity electric current to the upper anterior teeth according to the direction of the en-masse retraction movement (Fig. 3). The effectiveness and safety of proposed device were evaluated in a preliminary study conducted on six patients, which showed that the device was effective in accelerating orthodontic tooth movement without any adverse effects [36].

### Pain, discomfort, functional impairments, and satisfaction questionnaire

**Fig. 4:**
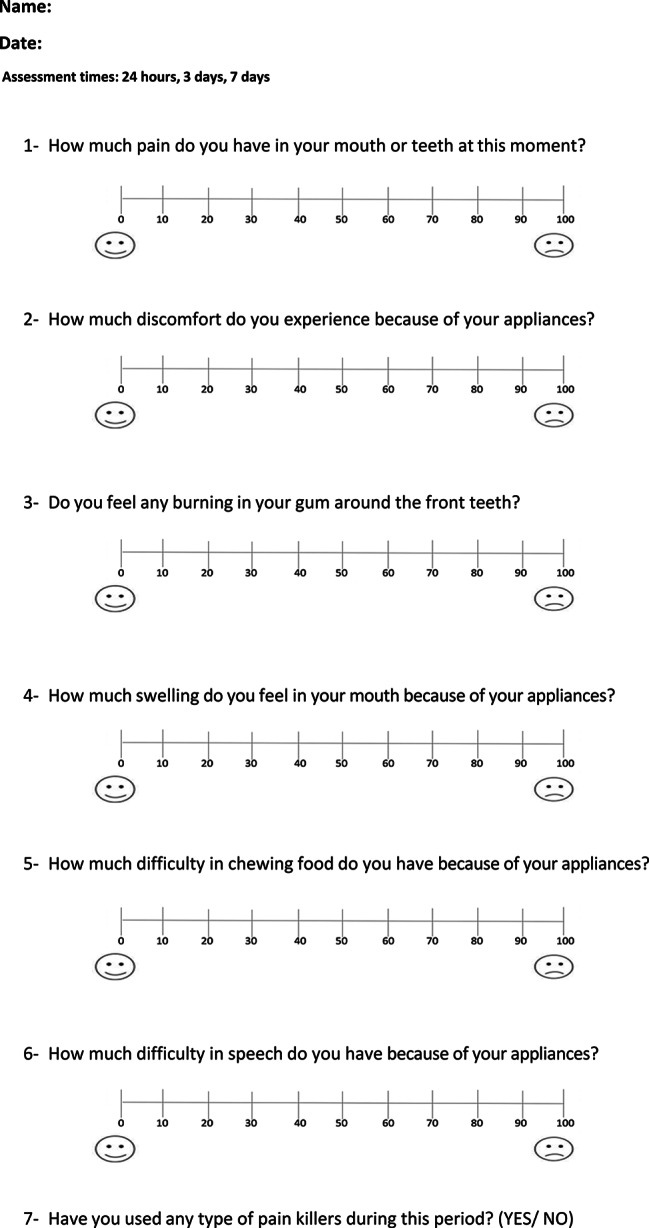
The First questionnaire that administered to patients in both group at (T1) 1st week of the 1st month 24 h after springs applied, (T2) 1st week of the 1st month 3 days after springs applied, (T3) 1st week of the 1st month 7 days after springs applied, (T4) 1st week of the 2nd month 24 h after springs re-activation, (T5) 1st week of the 2nd month after 3 days of spring re-activation, (T6) 1st week of the 2nd month after 7 days of spring re-activation, (T7) 1st week of the 3ed month after 24 h of spring re-activation, (T8) 1st week of the 3ed month after 3 days of spring re-activation, (T9) 1st week of the 3ed month after 7 days of spring re-activation

This trial utilized two questionnaires:

Questionnaires 1: This was distributed to patients in both groups to evaluate their perception of pain, discomfort and functional impairment at specific intervals: 24 h (T1), 3 days (T2), and after 7 days (T3) after the application of the retraction coil springs. Then, the same questionnaire was administered again in the second month after 24 h (T4), 3 days (T5), and 7 days (T6) of coil springs re-activation, and in the third month at the same times as before after the coil springs re-activation (T7,T8,T9). This questionnaire included questions about the following items: (1) pain levels, (2) discomfort levels, (3) burning sensation in the upper anterior teeth area, (4) swelling levels in the upper anterior teeth area, (5) chewing difficulties, (6) speech difficulties, (7) analgesics consumption (Fig. 4). Responses to these questions were collected using the Visual Analog Scale (VAS), except for question no 7 which had a dichotomous answer (yes/no). Based on the VAS scores, the severity of each variable was classified as follows: mild (less than 20), mild-to-moderate (from 20 to less than 40), moderate (from 40 to less than 60), moderate-to-severe (from 60 to less than 80), and severe (from 80 to 100) [11].

**Fig. 5:**
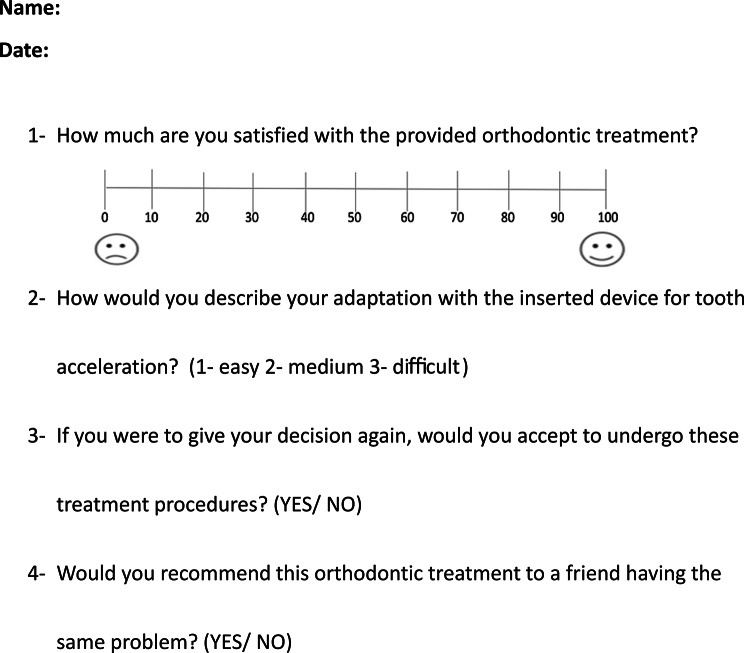
The second questionnaire administered to patients in both group at the end of the fifth month of the en-mass retraction

Questionnaire 2: This questionnaire was distributed at the end of the fifth month of the en-mass retraction in both groups, and included questions about the following items: (1) satisfaction with orthodontic treatment (2) willingness to undergo the treatment again, (3) recommending of this procedure to a friend. For the electrical current stimulation group (LIES group), Another question was added to this questionnaire about (4) the difficulty of adapting with the electrical device (Fig. 5). Patients’ responses to question 1 were collected using a VAS, while questions 2 and 3 required yes/no answers. The answers to question 4 were provided using a three-point scale: 1- easy, 2- moderate, 3- hard.

The questionnaire items were explained in a clear language close to patient’s understanding. Any inquiries from patients about the questionnaire were addressed by the researcher without affecting their responses. Patients experiencing moderate or severe pain were permitted to take Paracetamol Acetaminophen 500 mg, provided they completed the pain questionnaire first to ensure assessment accuracy.

### Statistical analysis

SPSS® Version 25 software (IBM, Armonk, NY, USA) was used to do the statistical analysis. The Shapiro-Wilk test was used to evaluate the Normality of data distribution. Independent sample t-test or its nonparametric alternative test (Mann–Whitney U test) were used to detect significant differences between the two groups. The Chi-square test was used to detect any significant differences between the two groups for the dichotomous variables. The Friedman’s test was used to verify the presence of significant differences in the studied variables over time, and the Cochran’s Q test to verify the presence of significant differences for the dichotomous variables over time. Bonferroni’s correction of the significance level was applied due to the multiplicity of pairwise comparisons, and the results of all tests were considered significant at *P* ≤ 0.005.

## Results

### Baseline sample characteristics

The sample consisted of 40 patients (8 males and 32 females) with mean age 21.1 ± 2.3 (17–25 years), twenty patients were distributed in each group. Over the course of the study, there were no patient dropouts, and all patients in both groups answered their questioners at all assessment points (Fig. 1). Age and gender did not significantly differ between the two groups (*P* = 0.612 and *P* = 0.429, respectively). The baseline sample characteristics regarding gender and age are given in Table 1.

**Table 1:** Basic sample characteristics

Variable		LIES group (*N* = 20)	CER group (*N* = 20)	Sample (*N* = 40)	*P*-value
		*n* (%)	*n* (%)	*n* (%)	
Gender	Female	17 (85.00)	15 (75.00)	32 (80.00)	0.429^a^
	Male	3 (15.00)	5 (25.00)	8 (20.00)	
Age	Mean ± SD	20.87 ± 2.17	21.24 ± 2.40	21.05 ± 2.27	0.612^b^

a: Chi-square test, b: independent sample t-test

LIES: low-intensity electrical stimulation, CER: conventional en-masse retraction, n: number of patients, SD: standard deviation

### Intergroup comparisons

#### Pain perception and the levels of discomfort

Patients in both groups experienced mild or mild to moderate pain at all follow-up times. Throughout all assessment times, the mean pain levels were smaller in the LIES group (between 0.15 ± 0.49 and 24.25 ± 8.26) compared to the CER group (between 1.95 ± 1.67 and 28.95 ± 8.06). However, statistically significant differences were only observed on the third and seventh days of the second and third months (*P* < 0.005, *P* = 0.001, *P* < 0.005, and *P* < 0.005 at T5, T6, T8, and T9 respectively; Table 2). The greatest mean pain values were recorded on the first day (T1) in both groups. Subsequently, the mean pain level decreased after 3, and 7 days (*P* < 0.001; Table 2). The same results were also noted after each activation of the retraction forces.

**Table 2:** Descriptive statistics of the levels of pain, discomfort, and difficulty in chewing perceived by the patients as well as the p-values of significance tests

Variable	Time point	LIES group (*N* = 20)			CER group (*N* = 20)			Mean Diff	95% CI of Difference Lower, upper	LIES vs. CER
		Mean ± SD	Median	*P*-value^c^	Mean ± SD	Median	*P*-value^c^			*P*-value
	T1	24.25 ± 8.26	24.25		28.95 ± 8.06	28.95		-4.70	-9.89, 0.49	0.075^a^
	T2	10.55 ± 5.38	10.55		15.45 ± 6.95	15.45		-4.90	-	0.008^b^
	T3	2.95 ± 3.44	2.95		5.90 ± 3.55	5.90		-2.95	-	0.009^b^
	T4	11.30 ± 6.76	11.30		16.20 ± 4.24	16.20		-4.90	-8.53, -1.27	0.010^a^
Pain	T5	3.50 ± 2.44	3.50	< 0.001**	8.60 ± 2.98	8.60	< 0.001**	-5.10	-6.85, -3.35	< 0.005^a^*
	T6	0.55 ± 0.76	0.55		2.85 ± 2.06	2.85		-2.30	-	0.001^b^*
	T7	10.05 ± 5.48	10.05		14.3 ± 3.26	14.30		-4.25	-	0.017^b^
	T8	2.90 ± 2.40	2.90		7.20 ± 2.14	7.20		-4.30	-	< 0.005^b^*
	T9	0.15 ± 0.49	0.15		1.95 ± 1.67	1.95		-1.80	-	< 0.005^b^*
	T1	37.85 ± 16.92	36.50		24.05 ± 8.72	22.00		13.80	5.09, 22.51	0.003^a^*
	T2	27.55 ± 12.50	30.00		14.15 ± 6.64	12.00		13.40	6.93, 19.87	< 0.005^a^*
	T3	18.75 ± 8.12	20.00		4.45 ± 2.86	5.00		14.30	10.32, 18.28	< 0.005^a^*
	T4	15.35 ± 4.91	15.00		13.90 ± 4.95	13.50		1.45	.	0.379^b^
Discomfort	T5	7.85 ± 2.37	7.00	< 0.001**	6.55 ± 3.14	6.00	< 0.001**	1.30	-0.48, 3.08	0.147^a^
	T6	1.85 ± 1.81	1.50		1.35 ± 1.39	1.00		0.50	.	0.444^b^
	T7	9.20 ± 4.51	10.00		8.70 ± 2.85	9.00		0.50	.	0.836^b^
	T8	3.95 ± 3.19	4.00		4.05 ± 2.80	5.00		-0.10	.	0.555^b^
	T9	0.80 ± 1.32	0.00		0.45 ± 0.89	0.00		0.35	.	0.336^b^
	T1	21.45 ± 9.27	20.00		26.80 ± 7.40	26.00		-5.35	-10.72, 0.02	0.051^a^
	T2	10.65 ± 5.91	9.00		14.95 ± 5.92	13.00		-4.30	.	0.015^b^
	T3	2.8 ± 3.33	1.00		5.00 ± 3.26	5.00		-2.20	.	0.038^b^
	T4	11.05 ± 5.07	10.00		14.25 ± 3.68	14.50		-3.20	-4.41, -0.79	0.006^a^
Chewing difficulty	T5	4.15 ± 3.45	4.00	< 0.001**	6.75 ± 2.00	7.00	< 0.001**	-2.60	.	0.027^b^
	T6	0.40 ± 0.99	0.00		1.10 ± 1.21	0.50		-0.70	.	0.038^b^
	T7	10.75 ± 3.89	10.00		10.45 ± 2.67	10.00		0.30	-1.84, 2.44	0.778^a^
	T8	3.20 ± 2.95	3.00		4.45 ± 2.04	4.00		-1.25	-2.87, 0.37	0.127^a^
	T9	0.20 ± 0.52	0.00		0.90 ± 1.52	0.00		-0.70	.	0.105^b^

a: Independent sample t-test, b: Mann-Whitney U test (*. significant at the 0.005 level.)

c: Friedman’s test (**. significant at the 0.05 level)

LIES: low-intensity electrical stimulation, CER: conventional en-masse retraction, SD: standard deviation, CI: confidence interval

T1: 1st week of the 1st month 24 h after springs applied T2: 1st week of the 1st month 3 days after springs applied T3: 1st week of the 1st month 7 days after springs applied T4: 1st week of the 2nd month 24 h after springs activation, T5: 1st week of the 2nd month after 3 days of spring activation, T6: 1st week of the 2nd month after 7 days of spring activation, T7: 1st week of the 3ed month after 24 h of spring activation, T8: 1st week of the 3ed month after 3 days of spring activation, T9: 1st week of the 3ed month after 7 days of spring activation

The discomfort levels were mild to moderate on the first day of en-masse retraction in the LIES and CER groups (mean VAS value = 37.85 ± 16.9, 24.05 ± 8.7, respectively). Then, the discomfort levels gradually decreased after 3, and 7 days (*P* < 0.001; Table 2). The discomfort level means were greater in the LIES group compared to the CER group at almost all follow-up times. However, significant statistical differences were only noticed during the first week of treatment (*P* = 0.003, *P* > 0.005, and *P* > 0.005, at T1, T2, and T3 respectively; Table 2).

#### Sensation of burning, swelling, chewing, and speech difficulties

Mild burning sensation was reported by patients in the LIES group at all follow-up times, with means scores ranged between 0.25 ± 0.64 to 1.80 ± 2.26. However, these levels of burning sensation were greater in the LIES group compared to the CER group. No significant statistical differences between the two groups except for the first and third days (T1, T2) of follow-up were noted (*P* = 0.001, *P* = 0.002, respectively; Table 3).

**Table 3:** Descriptive statistics of the levels of swelling, burning sensation, difficulty in speech, and satisfaction perceived by the patients as well as the p-values of significance tests

Variable	Time point	LIES group (*N* = 20)			CER group (*N* = 20)			Mean Diff	LIES vs. CER
		Mean ± SD	Median	*P*-value^c^	Mean ± SD	Median	*P*-value^c^		*P*-value
	T1	1.80 ± 2.26	0.00		0.00 ± 0.00	0.00		1.80	0.001^b^*
	T2	1.35 ± 2.25	0.00		0.00 ± 0.00	0.00		1.35	0.002^b^*
	T3	0.95 ± 1.82	0.00		0.00 ± 0.00	0.00		0.95	0.009^b^
	T4	0.40 ± 0.88	0.00		0.00 ± 0.00	0.00		0.40	0.038^b^
Burning	T5	0.35 ± 0.67	0.00	0.265	0.00 ± 0.00	0.00	-	0.35	0.019^b^
	T6	0.25 ± 0.64	0.00		0.00 ± 0.00	0.00		0.25	0.076^b^
	T7	0.30 ± 0.66	0.00		0.00 ± 0.00	0.00		0.30	0.038^b^
	T8	0.45 ± 0.83	0.00		0.00 ± 0.00	0.00		0.45	0.009^b^
	T9	0.30 ± 0.47	0.00		0.00 ± 0.00	0.00		0.30	0.009^b^
	T1	4.65 ± 3.63	5.00		5.65 ± 2.72	5.00		-1.00	0.342^b^
	T2	2.50 ± 3.71	0.00		1.25 ± 1.55	0.50		1.25	0.835^b^
	T3	0.10 ± 0.45	0.00		0.15 ± 0.49	0.00		-0.05	0.574^b^
	T4	0.00 ± 0.00	0.00		0.35 ± 0.81	0.00		-0.35	0.038^b^
Swelling	T5	0.00 ± 0.00	0.00	< 0.001**	0.00 ± 0.00	0.00	< 0.001**	0.00	1.0^b^
	T6	0.00 ± 0.00	0.00		0.00 ± 0.00	0.00		0.00	1.0^b^
	T7	0.00 ± 0.00	0.00		0.00 ± 0.00	0.00		0.00	1.0^b^
	T8	0.00 ± 0.00	0.00		0.00 ± 0.00	0.00		0.00	1.0^b^
	T9	0.00 ± 0.00	0.00		0.00 ± 0.00	0.00		0.00	1.0^b^
	T1	42.95 ± 17.98	39.00		3.75 ± 3.89	3.00		39.20	< 0.005^b^*
	T2	37.80 ± 16.36	34.00		0.70 ± 1.53	0.00		37.10	< 0.005^b^*
	T3	23.80 ± 11.75	19.50		0.00 ± 0.00	0.00		23.80	< 0.005^b^*
	T4	15.25 ± 3.93	15.00		0.00 ± 0.00	0.00		15.25	< 0.005^b^*
Speech difficulty	T5	12.90 ± 3.93	11.00	< 0.001**	0.00 ± 0.00	0.00	< 0.001**	12.90	< 0.005^b^*
	T6	11.45 ± 2.95	10.00		0.00 ± 0.00	0.00		11.45	< 0.005^b^*
	T7	9.75 ± 3.16	10.00		0.00 ± 0.00	0.00		9.75	< 0.005^b^*
	T8	8.65 ± 2.66	9.50		0.00 ± 0.00	0.00		8.65	< 0.005^b^*
	T9	8.10 ± 2.20	9.00		0.00 ± 0.00	0.00		8.10	< 0.005^b^*
Satisfaction		90.10 ± 7.38	90.00	-	87.35 ± 7.91	84.00	-	2.75	0.220^b^

a: Independent sample t-test, b: Mann-Whitney U test (*. significant at the 0.005 level), c: Friedman’s test (**. significant at the 0.05 level), LIES: low-intensity electrical stimulation, CER: conventional en-masse retraction, SD: standard deviation, CI: confidence interval

T1: 1st week of the 1st month 24 h after springs applied T2: 1st week of the 1st month 3 days after springs applied T3: 1st week of the 1st month 7 days after springs applied T4: 1st week of the 2nd month 24 h after springs activation, T5: 1st week of the 2nd month after 3 days of spring activation, T6: 1st week of the 2nd month after 7 days of spring activation, T7: 1st week of the 3ed month after 24 h of spring activation, T8: 1st week of the 3ed month after 3 days of spring activation, T9: 1st week of the 3ed month after 7 days of spring activation

Swelling sensation was at mild levels in both groups with no statistically significant differences between them in the first week of follow-up only (*P* > 0.005; Table 3). Then, no swelling was reported at any other evaluation times.

Mild to moderate chewing difficulty was initially reported 24 h after forces application (T1) with mean score 21.45 ± 9.27 in the LIES group and 26.80 ± 7.40 in the CER group. Then it was gradually decreased during the first week (*P* < 0.001; Table 2). Chewing difficulty means in the LIES group were smaller but not statistically significant compared to those in the CER group at all studied times, except for the first day of the third month (T7) where approximately similar means were found (*P* > 0.005; Table 2).

On the first day (T1) patients in the LIES group experienced moderate difficulty in speaking (mean VAS value = 42.95 ± 17.98), then it was significantly decreased to become mild at the end of follow-up (T9; mean VAS value = 8.10 ± 2.20; *P* < 0.001). Generally, the speech difficulty means were significantly greater in the LIES group compared to the CER group at all assessment times (*P* < 0.005; Table 3).

#### The consumption of painkillers and patients’ satisfaction and acceptance

The analgesics consumption amount was low in both groups, as only one patient in the LIES group, and two in the CER group reported that they have had pain killers (paracetamol 500 mg) on the first day only (T1), and did not differ significantly between both groups (*P* = 0.545).

The satisfaction levels were greater in the LIES group compared to that in the CER group with mean values 90.1, and 87.35, respectively. However, the difference was not significant (*P* = 0.220; Table 3).

The majority of patients in the LIES and CER groups (95% and 90%, respectively) revealed that they would recommend their friends and would accept to undergo the same treatment. The differences between both groups were insignificant (*P* = 0.548; Table 4).

**Table 4:** Descriptive statistics of treatment repeating and friend recommendation as well as the p-values of significance tests

		LIES group (*n* = 20)	CER group (*n* = 20)	LIES vs. CER
		*n* (%)	*n* (%)	*P*-value^a^
Treatment repeating	No	1 (5.00)	2 (10.00)	0.548
	Yes	19 (95.00)	18 (90.00)	
Friend recommendation	No	1 (5.00)	2 (10.00)	0.548
	Yes	19 (95.00)	18 (90.00)	

a: Chi-square test

LIES: low-intensity electrical stimulation, CER: **conventional** en-masse retraction, n: number of patients

#### Adaptation to the accelerating device

Twelve patients (60% of patients) in the LIES group found that the adaptation to the accelerating device was easy, compared to 40% who found it with moderately difficult. None of the patients found it difficult to habituate.

### Harms

No severe untoward effects were observed during the trial. However, only one patient in the experimental group reported an ulcer on the buccal mucosa under the buccal part of the device two days after starting to use the device. The ulcer completely disappeared after smoothing the edges of the device and postponing its use for seven days.

## Discussion

The objective of this trial was to evaluate the patient-reported outcome measures (PROMS) associated with the use of low-intensity electrical stimulation to accelerate the upper anterior teeth retraction in comparison with the conventional method. The success of orthodontic treatment depends on the use of techniques and devices that keep patients’ discomfort and pain as minimal as possible, since pain is one of the common problems that may affect patient cooperation and may lead to unfavorable treatment outcomes [39].

The mini-implant-assisted en-masse retraction technique was chosen in the current trial because it has been shown that better results can be achieved in this technique compared to the two-stage retraction technique with a TPA anchorage in terms of retraction velocity, dental changes, anchorage loss, and aesthetic treatment outcomes [40, 41]. No previous studies have evaluated the effect of low-intensity electrical stimulation on the pain caused by orthodontic treatment. Therefore, the results of the current work were compared with results of other studies that used physical methods of pain relief such as the transcutaneous electrical nerve stimulation (TENS) techniques.

Patients in both groups felt mild or mild to moderate pain during follow-up period. However, the mean pain level in the LIES group was smaller than that in the CER group at all assessment times, with statistically significant differences (*P* < 0.005) during the second and third months only. But, it noteworthy to know that these differences were not clinically significant. This may indicate unimportant clinical effect of the low-intensity direct electrical stimulation on orthodontic associated pain.

The effect of TENS on pain reduction was assessed in literature either after elastomeric separators placement [42, 43] or after the initial arch wire placement [44]. Generally, those previous studies’ results supported the efficacy of TENS in pain reduction during the first week after orthodontic procedures. Unlike the current study, where the results showed no statistically significant differences in pain scores during the first week of treatment between the two groups. On the other hand, when evaluating the results of other studies employing physical methods to relieve pain, such as low-level laser therapy (LLLT), LIES was less effective in reducing orthodontic pain. As the study of Bhat et al. reported significant relief in pain score when using LLLT with en-masse retraction. However, it noteworthy to know that the pain reduction was in one assessment time only (after 7 days), and the study had a split mouth design so that its results may not be reliable. Also, the studies of Almallah et al. and Owayda et al. reported that LLLT was effective way in controlling orthodontic pain after elastomeric separators placement [1, 45]. The maximum pain score was noted after 24 h of coil springs application in both groups, then it was gradually decreased after 3 and 7 days. These results agree with the results of the previous studies, which showed that pain usually peaks after 24 h of applying orthodontic force and then gradually decreases until it almost disappears after 7 days [9, 37]. The retraction forces re-activation in the second and third months made the pain increase again. However, it was also decreased along a week after activation.

In this study, the mean discomfort level in the experimental group was mild to moderate (37.85 ± 16.9) on the first day after device application, and then it gradually decreased during the observation period to become very mild (0.8 ± 1.32) after three months (*P* < 0.001). The use of the removable electrical device was the main reason for patients uncomfortable in the LIES group, as mentioned by patients, compared to the fixed appliances like in the CER group. This may be attributed to the mass and shape of the electrical device that covered the anterior region of the maxilla and caused some discomfort during daily activities. This result agrees with what was mentioned by previous studies of Idris et al. and Saleh et al. that evaluated the levels of discomfort associated with use of removable orthodontic appliances [5, 6].

The significant decrease in discomfort levels along the assessment times may be due to the patient’s habituation and adaptation to the applied devices. This was also consistent with previous studies about discomfort levels’ decreasing along with removable appliances treatment due to the adaptation [5, 6].

Regarding the burning sensation, statistically significant differences between the two groups were only noted on the first (T1) and third (T2) days of follow-up. Only small number of patients in the LIES group reported mild burning sensations sometimes while wearing the electrical device, especially after recharging the battery. This could be because of that the applied current voltage may become higher than 1.5 volts when the battery was fully charged and its voltage was too high. However, this burning sensation did not affect the safety of applying this electronic device or caused any side effects while it was being used. When reviewing the available literature, no previous studies evaluated this variable were found.

The observed mean swelling levels were mild in both groups, with no statistically significant differences between them. This swelling was noted only in the first few days of the follow-up, and may be attributed to the mucogingival irritation caused by the used retraction hooks and coil springs. This result agrees with the results of Mousa et al. study which evaluated patient-centered outcomes associated with the non-accelerated mini-implant-assisted en-masse retraction, as they reported mild swelling during the first week of the en-masse retraction [4].

Most patients in both groups experienced mild to moderate chewing difficulty after 24 h of forces application, then it gradually decreased during the first week to become mild or negligible. The retraction forces re-activation in the second and third months made the chewing difficulty increase again. However, it was also decreased along a week after activation. Although patients in the LIES group expressed lower levels of this discomfort, the differences were not significant between the two groups. This difficulty in food chewing could be mainly attributed to the elevated pain in the periodontal tissues caused by the applied orthodontic forces [37]. Therefore, less chewing difficulty was observed in the LIES group than the CER group, as patients in the LIES group expressed lower levels of pain compared to CER group. Also, the presence of mini-implants, hooks, and retraction coil springs inside the mouth increased the difficulty of chewing due to their interference with the patients’ cheeks and mucogingival membrane inside the mouth [4, 46]. Similar findings were reported in a compound design (parallel two groups with a split mouth design in each group) RCT conducted by Alfawal et al. about employing either piezocision or high-energy lasers in accelerating canines’ retraction, where mild to moderate chewing difficulty was observed on the first day of upper canines retraction in both experimental and control sides [24]. On the other hand, this finding disagrees with that of Mousa et al. study in terms of this difficulty severity where moderate to severe discomfort while eating was reported on the first day of the en-masse retraction by 68.4% of patients [4]. This may be due to the differences in the treatment protocols, as the premolars extraction and the mini-implants insertion were performed just before the start of the en-masse retraction in their study, unlike to the current study, where they were performed in the early stages of treatment, which gave more time for extraction sites recovery and habituation to the min-implants. Moreover, elastomeric chains were used in their study to apply the retraction forces, while nickel-titanium springs were used in this study, which apply light and continuous forces compared to elastomeric chain [47].

All patients in the LIES group reported some degree of speech difficulty while using the electrical accelerating device, which was moderate on the first day of the follow-up, then it slightly decreased by the end of the first week (T3) to become mild to moderate. Subsequently speech difficulty became very mild at the end of third month of follow-up (T9). This difficulty in speaking was actually expected due to the design of the device that covered the central incisors and the anterior region of the palate. Also, the mass of the device that interfered with the tongue spaces into the mouth [48, 49]. Despite this, a gradual decrease in the amount of speech problems was observed during the follow-up period. This could be attributed to the patient’s habituation to use the device after a while. This result was consistent with the results of Idris et al. and Saleh et al. studies which evaluated speech impairment with removable appliances. They found a statistically significant decline in speech impairment intensity during the treatment course [5, 6]. On the other hand, the main causes of speech difficulty in the control group were the orthodontic appliances component such as the hooks that were placed at the corners of the maxilla that might interfere with the patient’s lip, in addition to the retraction coil springs that might interfere with the patient’s cheek [4, 46].

Patients in both groups reported high levels of satisfaction with the treatment, with no statistically significant difference between them (*P* = 0.220). Also, the majority of them showed their willingness to repeat the treatment procedure again, and they would recommend their friends to undergo a similar treatment. This high percentage may indicate the ease of the procedure, and reflect the minor and tolerable pain and discomfort associated with this treatment which did not interfere with patients’ regular social activities. These results agree with what reported previously in a pilot study that conducted by Shaadouh et al. [36].

60% of patients in the LIES group found the adaptation to the accelerating device easy and 40% found it moderately difficult. None of patients reported significant difficulty with it. This finding agreed with the high acceptability of the treatment and demonstrated the ease of using the accelerating device.

The study sample included a greater number of females compared to males. However, after the random distribution of the sample into two groups, no differences were found between the two groups regarding the gender, which may rule out the influence of this factor on the results of this study. Unfortunately, the current study did not evaluate the effect of gender on the studied variables due to the small number of males compared to females, which makes any subgroup analysis to differentiate the responses between females and males less powerful from the statistical point of view.

On the other hand, although the orthodontic literature infrequently points to any correlation between gender and perception of pain during orthodontic treatment [7], some studies showed that there was a gender difference in response to pain [50, 51]. However, many other studies indicated that there were no statistically significant differences between the two sexes [52–54]. Thus, it is reasonable to believe that gender differences in pain behavior may reflect the influence of culture rather than differences in physiology [7].

### Harms

No associated harms or sever untoward effect were reported during the follow-up period, except for one patient who suffered from a small ulcer on the attached gingiva around the left central incisors two days after starting use the device, which was caused by an irritation from device’s edge. Despite of this, the appliance appeared safe during its use and this agrees with that of Shaadouh et al. about the safety of using low-intensity electrical stimulation and the electrical stimulation device [36].

### Limitations

One of the limitations of the current study was the inability to blind the researcher or the patients due to the nature of this study and the use of an electric device. Blinding was, therefore, confined to the outcome assessment only. A subgroup analysis to differentiate between male and female responses to the appliance was not performed. Another limitation of this study was the dependence on patient cooperation in wearing the removable electric stimulation device. This cooperation was not objectively measured during this trial. Therefore, the amount of tooth movement could have been affected if patients did not comply with the given instructions. Finally, this study focused only on patient-reported outcomes associated with the use of electrical stimulation during the en-masse retraction. Future studies are needed to assess other variables such as periodontal status, and teeth vitality after the use of these devices.

## Conclusions

Both the accelerated en-masse retraction by low-intensity electrical stimulation and the conventional en-masse retraction were accompanied by mild to moderate pain, discomfort, and chewing difficulty, and mild swelling sensation on the first day of retraction. The application of electric stimulation was associated with a negligible and transient burning sensation, but without any side effects.

The electrical acceleration device caused moderate difficulty in speech on the first day of using it, which continued but slightly decreased during the follow-up. Whereas, conventional en-masse retraction caused mild speech difficulty only on the first three days of treatment. The use of the electrical acceleration device was accompanied with high level of satisfaction and acceptance.

## Data Availability

All Data about the current study can be available from the corresponding author upon reasonable request.

## Abbreviations

LIES: Low-intensity electrical stimulation
CER: Conventional en-masse retraction
PAOO: Periodontally accelerated osteogenic orthodontics
LLLT: Low-level laser therapy
OTM: Orthodontic tooth movement
MET: Microcurrent electrical therapy
VAS: Visual analog scale
CONSORT: Consolidated Standards of Reporting Trials
NiTi: Nickel-titanium
SPSS: Statistical package for the social sciences
PROMS: Patient-reported outcome measures
TPA: Transpalatal arch
TENS: Transcutaneous electrical nerve stimulation
